# Preoperative statin is associated with decreased operative mortality in high risk coronary artery bypass patients

**DOI:** 10.1186/1749-8090-5-8

**Published:** 2010-02-24

**Authors:** James A Magovern, Robert J Moraca, Stephen H Bailey, David A Dean, Kathleen A Simpson, Thomas D Maher, Daniel H Benckart, George J Magovern

**Affiliations:** 1 Department of Cardiovascular and Thoracic Surgery, Allegheny General Hospital, Pittsburgh, PA, USA

## Abstract

**Background:**

Statins are widely prescribed to patients with atherosclerosis. A retrospective database analysis was used to examine the role of preoperative statin use in hospital mortality, for patients undergoing isolated coronary artery bypass grafting (CABG.)

**Methods:**

The study population comprised 2377 patients who had isolated CABG at Allegheny General Hospital between 2000 and 2004. Mean age of the patients was 65 ± 11 years (range 27 to 92 years). 1594 (67%) were male, 5% had previous open heart procedures, and 4% had emergency surgery. 1004 patients (42%) were being treated with a statin at the time of admission. Univariate, bivariate (Chi^2^, Fisher's Exact and Student's t-tests) and multivariate (stepwise linear regression) analyses were used to evaluate the association of statin use with mortality following CABG.

**Results:**

Annual prevalence of preoperative statin use was similar over the study period and averaged 40%. Preoperative clinical risk assessment demonstrated a 2% risk of mortality in both the statin and non-statin groups. Operative mortality was 2.4% for all patients, 1.7% for statin users and 2.8% for non-statin users (p < 0.07). Using multivariate analysis, lack of statin use was found to be an independent predictor of mortality in high-risk patients (n = 245, 12.9% vs. 5.6%, p < 0.05).

**Conclusions:**

Between 2000 and 2004 less than 50% of patients at this institution were receiving statins before admission for isolated CABG. A retrospective analysis of this cohort provides evidence that preoperative statin use is associated with lower operative mortality in high-risk patients.

## Introduction

The use of 3-hydroxy-3-methyl-glutaryl coenzyme A reductase inhibitors (statins) has been shown to reduce death, myocardial infarction and stroke in patients with elevated serum cholesterol and in those with near normal serum cholesterol levels [1]. The mechanism of this improvement is likely multifactorial, with some benefit attributed to lipid lowering effects and some to lipid-independent (pleiotropic) properties. Recently, evidence has accumulated that statins have beneficial effects on various portions of the clinical pathway that leads to atherosclerosis and cardiovascular events. These effects include downregulation of the inflammatory cascade [2], stabilization of the endothelial cell [3], attenuation of oxidative damage [4], decreasing thrombotic risk and possibly plaque stabilization [5]. The use of statins has steadily increased over time, but these drugs remain under utilized, relative to the larger population at risk for atherosclerosis. Patients who require coronary artery bypass grafting (CABG) represent a small segment of the entire population of patients with coronary artery disease. Many CABG patients have been treated with statins as outpatients before CABG, but a sizeable group present with no previous statin therapy. This study was undertaken to examine the efficacy of preoperative statin use on in-hospital mortality after CABG surgery.

## Patients and Methods

### Data Collection

Data were retrospectively abstracted from the institution's cardiac surgery database, which includes over 500 variables describing patient history, pre-, intra- and post-operative data and events and selected laboratory and functional testing results. Data from every patient who undergoes a major cardiac procedure is recorded on a standardized form and entered into the database by trained database staff during the admission and immediately following discharge. Data is collected under a waiver of consent from the Allegheny General Institutional Review Board.

### AGH Clinical Risk Score

As part of surgical consultation all patients are assigned a numerical clinical risk score (CRS) based on preoperative variables, including factors such as age, left ventricular function, comorbid diseases and laboratory studies. The risk score model is validated and has been described fully in a previous publication [6]. A recent study has confirmed the efficacy of the Magovern CRS in comparison to other well known risk assessment models [7]. The CRS ranges between 0 and 20 with a lower score predicting lower operative mortality. In this study, high risk patients were defined as those with a CRS of 9 or above (predicted mortality of at least 6%). Major postoperative morbidity was defined as the occurrence of any of the following complications: inotrope use for greater than 24 hours, acute myocardial infarction, cerebrovascular accident, respiratory failure, new onset renal failure, deep sternal wound infection or reoperation for bleeding/tamponade.

### Operative Technique

Standard anesthesia and surgical techniques were utilized for all patients. Based upon preoperative beliefs and pathology, patients were offered conventional cardiopulmonary bypass or when appropriate; an off pump technique. Conventional cardiopulmonary bypass utilized systemic heparinization (300 mg/kg heparin sulfate) to maintain an activated clotting time (ACT) >450 seconds. Off pump cardiac surgery utilized 100 mg/kg loading dose of heparin with a goal to maintain an ACT >300 seconds. Moderate hypothermia 34 Celsius was used in all cardiopulmonary bypass operations and patients were re-warmed to 36 Celsius prior to discontinuing the cardiopulmonary bypass. Cold blood cardioplegia was utilized for induction and maintenance.

### Patient Population

The study population comprised all patients who underwent isolated CABG surgery between January 1, 2000 and December 31, 2004. This study period was chosen because it afforded a large, comparable cohort of patients in both the statin and non-statin treated groups. Patients were considered to be in the "statin" group if they were taking statins at the time of admission or they were started during the admission prior to surgery. No data regarding the specific drug or dose was obtained.

### Statistical Analysis

Data are expressed as mean ± standard deviation (continuous variables) or incidence and percent of the relevant group. The study endpoint was defined as all cause in-hospital mortality. Bivariate analyses were performed to examine associations between preoperative status variables and this endpoint, using Student's t-test, Mann-Whitney rank sums test, Chi-square analysis and Fisher's exact test, as appropriate for continuous and dichotomous variables. Variables with significant (p < 0.15) bivariate evidence of association with the endpoint were then evaluated using stepwise logistic regression to determine which of the variables contributed to a multivariate prediction model for in-hospital mortality. BMDP Statistical Software, release 7 was used to perform all analyses.

## Results

Both the proportion of preoperative statin usage and the predicted mortality score for coronary bypass patients were similar over the course of the study period (Table 1). Table 2 illustrates the prevalence of relevant pre- and intra-operative variables in the overall population and in the statin and no statin study groups. There was no difference in incidence of any major preoperative co-morbidity. Mean left ventricular ejection fraction (LVEF) was 47.4 ± 13.8 in the no-statin group and 48.6 ± 13.6 in the statin group. Distribution of LVEF values (0-19%, 20-29%, 30-39%, 40-49%, 50% and higher) was also similar. The no-statin group was statistically older from the statin group (65.4 vs. 64.8, p = 0.02), however this difference has negligible clinical relevance.

**Table 1:** Preoperative statin usage and clinical risk score in CABG patients by year of surgery

Year:	2000	2001	2002	2003	2004
Statin Usage (%)	246/647 (38%)	220/523 (42%)	204/447 (46%)	181/423 (43%)	153/337 (45%)
Mean CRS	4.6 ± 3.7	4.5 ± 3.2	4.7 ± 3.7	4.0 ± 3.0	4.5 ± 3.2

CRS: Allegheny General Hospital cardiac surgery clinical risk score.

**Table 2:** Study Population and results of bnivariate analysis of statin groups

	All	Statin	No Statin
Patients	2377	1004	1373
Age (years)	65.4 ± 10.7	64.8 ± 10.9	65.8 ± 10.3^a^
Off-Pump CABG	321 (13.4%)	167 (6.9%)	154 (6.4%)
CAB Grafts	2.9 ± 1.0	2.8 ± 1.0	2.9 ± 1.1
AXC (minutes)	72.4 ± 25.1	73.0 ± 25.3	71.9 ± 24.9
CPB (minutes)	103 ± 33	104 ± 33	102 ± 32
Non-elective procedure	1376 (57%)	568 (57%)	808 (59%)
Redo Procedure	125 (5.3%)	66 (6.6%)	59 (4.3%)^b^
Female gender	719 (30.2%)	298 (29.7%)	421 (30.7%)
Congestive heart failure history	316 (13.3%)	133 (13.2%)	183 (13.3%)
Cardiomegaly	94 (4.0%)	41 (4.1%)	53 (3.9%)
Atrial arrhythmia history	133 (5.6%)	43 (4.3%)	87 (6.3%)
Body mass index < 25	340 (14.3%)	132 (13.1%)	208 (15.1%)
Preoperative AMI	428 (18.0%)	167 (16.6%)	261 (19.0%)
COPD	402 (16.9%)	184 (18.3%)	218 (15.9%)
Peripheral vascular disease	340 (14.3%)	144 (14.3%)	196 (14.3%)
Stroke history	144 (6.1%)	56 (5.6%)	88 (6.4%)
Renal failure	77 (3.2%)	28 (2.8%)	49 (3.6%)
Tobacco past/current	1101 (46.3%)	499 (49.7%)	602 (43.8%)
IDDM	260 (10.9%)	124 (12.4%)	136 (9.9%)
In-hospital death	56 (2.4%)	17 (1.7%)	39 (2.8%)^c^

AMI: acute myocardial infarction, a: *p *= 0.02; b: *p *= 0.01; c: *p *= 0.07.

Patients undergoing a redo open heart procedure were more likely to be receiving statin treatment, likely reflecting more focused cardiac care following the previous surgery. Surgical indices of grafts performed, and cross-clamp and cardiopulmonary bypass times did not differ between groups. 13.4% of patients underwent off-pump CABG, with no difference noted between the statin and non-statin groups. There were no differences regarding intubation time, ICU duration and hospital stay. The results of bivariate analysis between mortality rate and relevant preoperative variables are shown in Table 3. Each of these variables, with the exception of non-insulin diabetes mellitus, was used for the multivariate assessment. Multivariate analysis was used to determine which variables were independently associated with decreased mortality in the study population. Variables with a significant independent contribution to mortality risk are illustrated in Table 4. When considering the entire population there was no significant contribution of statins to operative risk. However the absence of statin treatment was associated with an increase in mortality in the subset of high risk patients with an estimated risk of mortality of 6% and greater.

**Table 3:** Results of bnivariate analysis of mortality rates associated with selected preoperative variables

Variable:	Present	Not Present	*p *value
Preoperative statin	17/1004 (1.7%)	39/1373 (2.8%)	0.07
Preoperative AMI	19/428 (4.4%)	37/1949 (1.9%)	0.002
Preoperative anemia	18/437 (4.1%)	38/1940 (2.0%)	0.007
Body mass index < 25	14/340 (4.1%)	42/2037 (2.1%)	0.02
Non insulin dependent diabetes	10/500 (2.0%)	46/1877 (2.5%)	0.55
Insulin dependent diabetes	14/260 (5.4%)	42/2117 (2.0%)	0.006
Chronic obstructive pulmonary disease	16/402 (4.0%)	40/1975 (2.0%)	0.02
Stroke history	6/144 (4.2%)	50/2233 (2.2%)	0.14
Peripheral vascular disease	12/340 (3.5%)	44/2037 (2.2%)	0.12
Renal failure history	5/77 (6.5%)	51/2300 (2.2%)	0.02
Atrial arrhythmia history	14/133 (10.5%)	42/2244 (1.9%)	< 0.001
Congestive heart failure history	21/316 (6.7%)	35/2061 (1.7%)	< 0.001
Tobacco past/current	33/1101 (3.0%)	23/1276 (1.8%)	0.06
Female gender	25/719 (3.5%)	31/1658 (1.9%)	0.02
Cardiomegaly	8/94 (8.5%)	48/2283 (2.1%)	< 0.001
Redo procedure	9/125 (7.2%)	47/2252 (2.1%)	< 0.001
	Survivors	In-hospital death	
Age (years)	65.3 ± 10.7	71.5 ± 8.9	< 0.001

AMI: acute myocardial infarction.

**Table 4:** Preoperative variables identified as independent predictors of in-hospital mortality following CABG surgery

	Coefficient	*p *value
All Patients		
Atrial arrhythmia history	0.84	< 0.001
Congestive heart failure history	0.87	< 0.001
Age (years)	0.06	< 0.001
Redo procedure	1.20	0.009
Preoperative AMI	0.84	0.005
Insulin dependent diabetes	0.85	0.009
Tobacco past/current	0.77	0.024
Female gender	0.56	0.066
High Risk Patients		
Insulin dependent diabetes	1.10	0.070
Preoperative statin use	-1.07	0.030

AMI: acute myocardial infarction.

Multivariate regression analysis was also performed using composite postoperative major morbidity as an endpoint. Statin usage was not shown to have a significant impact on composite major morbidity in this limited assessment.

## Discussion

Statins are one of the most effective medicines introduced in the past 25 years. Nonetheless they are still relatively under prescribed, especially in patients without symptomatic or obvious atherosclerosis and those without severe hypercholesterolemia. Recently, our knowledge regarding the biology of the non-lipid lowering, or pleiotropic effects of statins has rapidly expanded. Simultaneously, a number of recent reports have suggested a salutary effect of statins on perioperative mortality for patients undergoing CABG.

Clark, et al, reported a retrospective database study from the Medical University of South Carolina covering 3829 patients between 1996 and 2002 [8]. Only 1044/3829 patients received preoperative therapy (28%). In a propensity matched analysis they demonstrated significant association between preoperative statin therapy and lower 30 day mortality and morbidity. These findings paralleled those of an earlier study by Pan et al from the Texas Heart Institute [9]. This study evaluated 1563 patients who underwent CABG with CPB at a single institution. Multivariate analysis was used to show a 50% reduction in the risk of perioperative (30 day) death in those patients who received statins preoperatively. The use of statins preoperatively was not associated with a lower incidence of post-operative complications. In a propensity matched subgroup analysis statin therapy was associated with a significantly lower risk of the composite endpoint including death and stroke (but not death alone).

Collard, et al, showed similar results in a large international, multi-institutional study [10]. The primary study was a longitudinal analysis of 5436 patients at 70 centers undergoing CABG. The statin study was a post-hoc retrospective analysis using this database and showed reduced early cardiac mortality in patients receiving statins who underwent elective CABG (0.3% vs. 1.4%). Further, the discontinuation of statins post-operatively was associated with increased all-cause hospital mortality (2.6% vs. 0.6%) compared to those who had statin therapy maintained.

Statin use in the current study averaged 42% (range 38 - 46%) over the five-year period of 2000-2004. The relatively low prevalence may represent a referral bias in that our center is a primary angioplasty referral center. Consequently, many patients have a new diagnosis of coronary artery disease and the statin is not always started before operation, especially in the urgent or emergent situation. Beginning in 2005 our isolated CABG population demonstrated an increase in preoperative statin use to greater than 80%.

The most important objective of this study was to determine if the use of preoperative statin therapy is associated with reduced postoperative mortality. In each of the 5-years of the study, the mortality rate was lower in the group of patients exposed to statins preoperatively (range 26 - 60%.) In total, the net effect was to reduce mortality from 2.8% to 1.7%. The effect was seen in all groups, but was most notable in the high risk cohort (12.9% vs. 5.6%, p < 0.05), where the predicted mortality was 6% and higher.

This study was not designed to explain how statins exert their salutary effect. Nonetheless, a number of hypotheses are generated. Our group [11] and others [12] have recently shown that patients undergoing heart surgery who have elevated risk based on standard preoperative variables (age, left ventricular dysfunction, co-morbid disease) have evidence of ongoing inflammation manifested by elevated levels of inflammatory mediators such as interleukin-6 (IL-6) and C-reactive protein (CRP). Statins have been shown to ameliorate the inflammatory cascade in a number of models and these properties may confer protection from the inflammatory response induced by open heart surgery. This may explain the more pronounced protective effect of statins in our high risk cohort.

Statins have also been shown in clinical trials to be associated with decreased mortality when administered in the first 24 hours after acute myocardial infarction [13,14]. This may be the result of their potential ability to limit infarct size, as demonstrated in animal models of acute infarction [15]. These properties may contribute to the beneficial effect associated with CABG and would help to explain the more marked effect in high risk patients, many of whom require urgent or emergent surgery in the setting of acute ischemia or infarction.

The current study, in addition to all the other studies on preoperative statin use, is retrospective and nonrandomized. This introduces the issue of selection bias and other confounding variables. The statistical analysis minimizes this possibility, but it cannot eliminate this issue completely. Further, we do not have specific information regarding the specific statin, dose, duration or cholesterol lowering efficacy. There may be important factors with regard to dosage and duration of therapy that impact the benefit of statins in this patient population that we can not identify with this study. Nonetheless, we have shown a consistent reduction in perioperative mortality in patients being treated with statins, particularly those with elevated operative risk. While placebo controlled trials will likely not be possible, further study of the underlying mechanisms of these effects are needed.

## Competing interests

The authors declare that they have no competing interests.

## Authors' contributions

JAM performed the initial study design and oversight of the manuscript preparation. RJM contributed to the statistical analysis, designed and wrote the tables and performed all the major and minor revisions of the manuscript. SAB assisted in study design, manuscript preparation, and presentation at national meeting. DAD assisted in study design and statistical analysis. KAS developed the database and performed statistical analysis. DHB assisted in study design and manuscript preparation. TDM assisted in manuscript preparation, initial data analysis and study design. GJM Jr. performed the initial study design and authored key sections of the manuscript. All authors read and approved the final manuscript.
